# Obesity and Serious Mental Ill Health: A Critical Review of the Literature

**DOI:** 10.3390/healthcare2020166

**Published:** 2014-04-01

**Authors:** Tim Bradshaw, Hilary Mairs

**Affiliations:** Mental Health Nursing, School of Nursing, Midwifery and Social Work, University of Manchester, Oxford Road, Manchester, M13 9PL, UK; E-Mail: hilary.j.mairs@manchester.ac.uk

**Keywords:** serious mental illness, obesity, physical health

## Abstract

Individuals who experience serious mental ill health such as schizophrenia are more likely to be overweight or obese than others in the general population. This high prevalence of obesity and other associated metabolic disturbances, such as type 2 diabetes and cardiovascular disease, contribute to a reduced life expectancy of up to 25 years. Several reasons have been proposed for high levels of obesity including a shared biological vulnerability between serious mental ill health and abnormal metabolic processes, potentially compounded by unhealthy lifestyles. However, emerging evidence suggests that the most significant cause of weight gain is the metabolic side effects of antipsychotic medication, usual treatment for people with serious mental ill health. In this paper we review the prevalence of obesity in people with serious mental ill health, explore the contribution that antipsychotic medication may make to weight gain and discuss the implications of this data for future research and the practice of mental health and other professionals.

## 1. Introduction

Obesity has been described as a global epidemic increasingly affecting populations in both developed and developing world countries alike [1]. In the United Kingdom it has been estimated that up to 61.3% of adults are overweight or obese. The current cost of treating obesity related health problems is approximately £5 billion per year, a figure set to double by 2050 [2]. Common health problems related to obesity include a higher risk of developing type 2 diabetes, heart disease and some cancers [1].

Individuals with serious mental ill health (SMI) such as schizophrenia are even more likely to be overweight or obese than other members of the general population [3]. For example one recent North American study found nearly 80% of a sample of over 10,000 people with diagnoses of schizophrenia, bipolar disorder or depression to be overweight or obese [4].

The reason for this high prevalence of obesity in people with SMI has been a source of much debate. Some have argued that weight gain in SMI is due to a complex interaction between genetic factors, environment, the mental illness itself and the effects of antipsychotic medication [5]. Whilst others lay the blame for weight gain more firmly on the side effects of antipsychotic medication [6,7,8,9]. This debate is further complicated by the potential effects of the unhealthy lifestyles (e.g., increased rates of smoking and reduced activity levels) that many individuals with SMI lead [10,11].

The aim of this paper was to undertake a broad review of the literature which examines issues related to obesity in people with SMI. We reviewed evidence regarding the prevalence of obesity in SMI, its possible causes and its effects on physical health. We then considered literature regarding potentially efficacious interventions to manage weight gain before discussing the implications of the review for future research and clinical practice. In order to advance knowledge beyond the mental health field one of our key aims was to make the paper accessible and relevant to other health professionals who may be involved in the physical health care people with SMI.

## 2. Search Strategy

A broad search of the literature was conducted up to and including December 2013 using several electronic databases including Medline, PsycINFO and EMBASE. Search terms used included combinations of “serious mental illness”, “severe mental illness”, “psychoses” or “psychotic disorder”; “weight gain”, “obesity”, “metabolic effects” or “causes of weight gain”; “physical health”, “medical co-morbidity”, “mortality” or “metabolic syndrome”; “antipsychotic medication”, “neuroleptic medication” or “psychotropic medication”; “weight management” or “lifestyle intervention”.

## 3. Inclusion/Exclusion Criteria

Consistent with the aims of the review we adopted a broad criteria including papers about weight gain and obesity in SMI, the effects of this weight gain on physical health, causes of weight gain and weight management interventions targeted at people with SMI. Due to translation issues we excluded non English language publications.

## 4. What Is Serious Mental Ill Health and How Is It Treated?

In the context of this paper serious mental ill health (SMI) refers to a range mental health disorders such as schizophrenia, schizoaffective disorder, bipolar disorder and psychotic depression. 

Symptoms of these disorders include positive psychotic symptoms such as hallucinations (e.g., hearing voices when no one is around), delusions (e.g., strange beliefs or fears which are not in keeping with the person’s culture) and/or thought disorder (e.g., disorganized or none intelligible speech patterns) [12,13]. Such symptoms are often the cause of significant distress and disruption to the lives of those who experience them and may lead them to come into contact with mental health services. People with SMI may also experience negative symptoms which manifest as behavioural deficits such as restricted expression of thoughts and feelings, lack of pleasure in normally enjoyable activities and avolition [12]. The latter often adversely affects self care and quality of life [14]. They may also experience disturbances in mood, including episodes of depression and mania.

Standard treatment for SMI usually includes the prescription of antipsychotic medication either one of the older or first generation drugs or increasingly one of the newer second generation preparations (see Table 1 for an overview of the different types of antipsychotic drugs). First generation drugs were introduced in the 1950s following the development of Chlorpromazine [15]. These drugs were thought to work by blocking the uptake of dopamine by synapses in the brain. Unfortunately they also inhibited the uptake of other neurotransmitters which resulted in a range of adverse effects such as extrapyramidal (muscle stiffness, involuntary movements, *etc*.) and anticholinergic (dry mouth, blurred vision, *etc*.) side effects. The second generation antipsychotic medications were developed and marketed in the early 1990s and were said to have a much lower extrapyramidal side effect profiles although many of the studies that concluded this were industry sponsored and more recently independent trials have called this assertion into question [16].

**Table 1:** Commonly used antipsychotic drugs (trade name).

First generation antipsychotics	Second generation antipsychotics
Chlorpromazine (Largactil)	Amisulpride (Solian)
Haloperidol (Haldol)	Aripiprazole (Abilify)
Zuclopenthixol Dihydrochloride (Clopixol)	Risperidone (Risperdal)
Prochlorperazine Maleate	Olanzapine (Zyprexa)
(Compazine, Stemzine, Buccastem, Stemetil, Phenotil)	Quetiapine (Seroquel)
Trifluoperazine (Stelazine)	Ziprasidone (Geodon)
Flupenthixol (Depixol)	Clozapine (Clozaril)
Clopenthixol (Sordinol)	

More recent attention has turned to a different side effect—that of medication induced weight gain. While the prescription of antipsychotic medication has long been associated with weight gain in people with SMI [8] recent evidence suggests weight gain is often much more rapid for the second generation medications [9].

## 5. Obesity in SMI

The findings of four national studies examining the prevalence of obesity and other cardio metabolic risk factors in people with SMI are represented in Table 2. The studies were conducted in Australia [17], North America [4] and continental Europe [18,19]. The data shows the percentage of individuals classified as overweight or obese according to body mass index (overweight = BMI ≥ 25 kg/m^2^; obese = BMI ≥ 30 kg/m^2^) range from 46% [18] to 79% [4]. In each of the studies significantly more individuals with SMI were overweight or obese compared to others in the general population. The studies also show a higher prevalence of other cardio-metabolic risk factors such as hypertension, elevated triglycerides levels and abnormally high levels of metabolic syndrome for groups with a relatively young average age. Metabolic syndrome is diagnosed by the presence of three out of the following five metabolic abnormalities abdominal obesity, elevated blood pressure, elevated fasting plasma glucose, high serum triglycerides, and low high-density cholesterol (HDL) level. It has been associated with a significant increase in the risk of developing cardiovascular disease and diabetes and normally becomes more prevalent with age [20]. Correll *et al*. (2010) [4] found a metabolic syndrome prevalence of 52% in an SMI group with a mean age of 44.7 years compared to only 20%–25% of adults in the U.S. general population in the same age bracket [21].

**Table 2:** National surveys of obesity in serious mental ill health (SMI).

Study	Design	Participants	Body Mass Index (BMI)	Other metabolic risk factors	Metabolic syndrome
Galletly *et al*. (2012) [17] Australia	National survey of psychosis	*n* = 1825 with a diagnosis of psychosis, 59.5% male, mean age was 38.36 (SD 11.16) years	29.1% overweight, 46.4% obese prevalence of obesity higher in women (52.5%) than men (42.4%).	47.2% hypertensive 66.6% smoked 96.7% low or very levels of physical activity 48% elevated triglycerides	54.8%
Correll *et al*. (2010) [4] USA	National metabolic screening programme	*n* = 10,084, 43% male, mean age 44.7 (SD 11.8) years, 55% white All patients had a self reported diagnosis of schizophrenia, bipolar disorder, or depression.	27% overweight, 52% obese	Mean waist circumference 41.1 inches in men and 40.4 inches in women 51% hypertensive 51% elevated triglycerides	52%
Limosin *et al*. (2008) [18] France	National survey of people with a diagnosis of schizophrenia	*n* = 5756, 62.8% male, mean age 37.1 (SD 11.8) years	29% overweight, 17% obese	Not specified	Not specified
Bernardo *et al*. (2009) [19] Spain	National cross sectional survey	*N* = 733, 71.7% male, mean age 37.8 (SD 11.3) years	Mean BMI = 26.7 men and 27.9 in women	71% smoked 18% hypertension	24%

The evidence reviewed shows that individuals with SMI are significantly more likely to be overweight or obese than other members of the populations in which they live and that this may also be associated with other metabolic disturbances.

## 6. Physical Health of People with Serious Mental Ill Health

There is compelling evidence that many people with SMI experience poorer physical health compared to others in the general population and that this results in reduced life expectancy of up to 25 years [22]. Whilst some of this excess premature mortality is from unnatural causes of death such as suicide most of it results from natural causes which may be related to the high levels of obesity and metabolic syndrome illustrated in Table 2. For example, Brown *et al*. (2010) [23] have been studying mortality in a cohort of 370 community dwelling individuals with a diagnosis of schizophrenia living in the South of England. In their latest 25 year follow up 164 (44%) had died. The mean age at death was 57.3 years for males and 65.5 years for females; both being almost 20 years lower than expected. In 81% of cases death resulted was from natural causes the most common being from circulatory disease which accounted for 33% of all observed mortality. Other studies have also shown similar findings for example, a systematic review [24] reported that individuals with SMI lost 20% of life expectancy with more than two thirds of them dying of coronary heart disease, compared with only approximately one half of deaths in the U.S. general population.

## 7. Causes of Obesity in People with Serious Mental Ill Health

Reasons that have been suggested for the high prevalence of obesity in people with SMI include lifestyle factors, mental illness, genetics, side effects of anti-psychotic medication or possibly a complex interaction of all of these factors [5].

### 7.1. Lifestyle and SMI

Individuals with SMI have been shown to lead unhealthy lifestyles compared to others [25,26]. McCreadie *et al*. (2003) [11] assessed the lifestyles of 102 community dwelling patients with schizophrenia and found them to consume diets that were high in saturated fats and low in fibre, 70% smoked and 70% were found to be overweight/obese. Using the Framingham assessment of cardiovascular risk the mean 10 year risk of coronary heart disease was higher in both males and females with SMI but this was only significantly so in males [11].

Henderson *et al*. (2006) [27] studied the dietary intake of 88 community dwelling individuals with schizophrenia comparing them to data from a national survey of the U.S. general population. The participants were significantly more likely to be overweight or obese and the nutritional content of their diets were poorer. However participants actually consumed significantly fewer calories per day than members of the general population leading the authors to conclude that obesity in SMI is not solely related to food consumption but may be due to other factors including medication side effects and lower levels of physical activity.

The findings of the studies reviewed above suggest that many people with SMI do indeed lead unhealthy lifestyles, eating diets that are poorer in nutritional content, smoking more and engaging in less physical activity than they should do. Obviously such unhealthy lifestyle factors are likely to have a detrimental effect on physical health however the study by Henderson *et al*. (2006) [27] suggests that the weight problems experienced by this client group may not necessarily be solely as a result of over eating and other factors should be considered.

### 7.2. Genetics

Genetics may have a role to play in increasing the vulnerability of some individuals towards developing SMI [28]. For example all of us have approximately a 1% lifetime risk of experiencing an episode of schizophrenia whereas for those with a first degree relative who has the illness this risk increases to approximately 10% [29]. Genes also play an important role in vulnerability to metabolic disorders such as obesity and diabetes and it has been suggested that a possible shared genetic link may explain the association between these disorders [30,31]. In an attempt to explain the association between obesity and SMI further Holt and Peveler (2009) [5] explored the processes which influence appetite stimulation, satiation and weight control in individuals with and without SMI concluding that *“the development of obesity in SMI results from a complex interaction of the genotype and environment of the person with mental illness, the mental illness itself and anti-psychotic medication” (page 665).* It should be noted however that the suggestion of a shared genetic link between obesity and SMI remains speculative and is not empirically supported. Indeed Moncrieff (2013) [32] argues that many such papers have been written by academics with links to the pharmaceutical industry and the attempt to suggest a link between SMI and metabolic disorders was in order to protect the reputation of their products. In 2006 the New York Times [33] printed an article about how Eli Lilly manufacturers of Olanzapine (Zyprexa) had engaged in a decade long effort to play down its risks to health. Documents supplied by a lawyer representing patients who claimed to have developed diabetes due to taking the drug were said to show how Lilly executives had *“kept important information from doctors about Zyprexa’s links to obesity and its tendency to raise blood sugar”* [33]*.*

### 7.3. Effect of Antipsychotic Medication on Weight Gain

The findings of three reviews and meta-analysis of data regarding weight gain resulting from taking antipsychotic medication have been presented in Table 3 [6,8,9]. The data shows that whilst all antipsychotic agents have the potential to cause weight gain when compared with placebo (where participants lost 0.74 kg in weight [8]) the first generation antipsychotics generally cause less weight gain at least in the shorter term than the newer second generation products. Two of the second generation drugs in particular Olanzapine and Clozapine may result in most rapid weight gain in the early stages of treatment, with data suggesting an increase of up to 17 kg in the first year of treatment [9]. Furthermore Foley and Morley (2011) [9] suggest that methodological limitations in the studies such as failing to assess for the effects non compliance with medication and conservative effects of using last observation carried forward (LOCF) to account for missing data means that actual weight gain could be underestimated by up to 50%.

There is also a paucity of longitudinal studies which have evaluated whether weight gain continues beyond 12 months. Only one study by Addington *et al*. (2006) [34] followed up a cohort of 189 first episode patients over three years. The authors found that weight gain was most significant in the first 3 months of treatment (5.3%), then 12% after one year, this began to slow between 12–24 months (2.3%) but continued at this slower rate between 24–36 months (2.2%). Over 3 years there was no change in weight for 8% of the sample, 32% gained weight and then lost it later with 18% returning to baseline levels. However, for 60% there was continued weight increase over the three years of an average 27% of their baseline weight (mean BMI = 31). The authors do not identify which drugs caused the most weight gain or the characteristics of the 60% who continued to gain weight, however the findings suggest that some individuals may be particularly vulnerable to medication induced weight gain. It is also possible that this weight gain may continue at the slower rate of approximately 2% each year beyond the 3 years reported, however currently there is no data to support or refute this. Further research is required to assess the impact of medication induced weight gain in the longer term. Additional evidence regarding the percentage of people affected by antipsychotic weight gain comes from Khan *et al*. (2008) [35] multi-site European study. The authors randomised 498 people with SMI to receive one of five different antipsychotic medications. After one year of treatment over half of all participants had gained weight; the most significant being for those prescribed Olanzapine who gained an average of 13.9 kg, with 71 out of 83 (86%) gaining more than 7% of their baseline body weight. The number prescribed Olanzapine classified as being overweight rose from 16% at baseline to 54% after one year. Furthermore as 30 (33%) of those prescribed Olanzapine were non compliant with the medication it seems likely that the amount of weight gain reported is likely to have been underestimated.

**Table 3:** weight gain associated with both first and second generation antipsychotic medication.

Study	Description	Medication	Comments
Allinson *et al*. (1999) [8]	Comprehensive Research Synthesis and meta analysis of 81 studies estimating weight change after 10 weeks of treatment at standard dose	Placebo = −0.74 kg	
		Molindone = −0.39 kg	
		Thioridazine = +3.19 kg	
		Ziprasidone = +0.04 kg	
		Risperidone = +2.10 kg	
		Sertindole = +2.92 kg	
		Olanzapine = +4.15 kg	
		Clozapine = +4.45 kg	
Alvarez-Jimenez *et al*. (2008) [6]	Meta analysis of data from 51 RCT’s including data on short term weight gain <9 months and long term weight gain ≥9 months for both chronic psychotic and first episode patients	**Chronic patients**	Dearth of RCTs reporting high quality data regarding long term weight gain, 8 studies for chronic patients and 4 studies for first episode patients
		***Short term weight gain***	
		Haloperidol = +0.01–1.4 kg	
		Risperidone = +1.0–2.3 kg	
		Olanzapine = +1.9–5.4 kg	
		***Long term weight gain***	
		Haloperidol = −0.7 to +0.4 kg	
		Risperidone = +0.4–3.9 kg	
		Olanzapine = +2.0 to 6.2 kg	
		**First episode psychosis**	Weight gain was 3- to 4-fold greater in studies that included young patients with limited previous exposure to antipsychotic
		***Short term weight gain***	
		Haloperidol = +2.6 to 3.8 kg	
		Risperidone = +4.0 to 5.6 kg	
		Olanzapine = +7.1 to 9.2 kg	
		***Long term weight gain***	
		Haloperidol = +4.0 to 9.7 kg	
		Risperidone = +6.6 to 8.9 kg	
		Olanzapine = +10.2 to 15.4 kg	
**Study**	**Description**	**Medication**	**Comments**
Foley and Morley (2011) [9]	Meta analysis of data from 25 studies of people with first episode psychosis who were either antipsychotic naive (*n* = 20) or had only been exposed to antipsychotics for between 9 days and 16 weeks (*n* = 5)	**By 6 to 8 Weeks**	Lower pre-treatment BMI younger age, triglyceride level, more negative symptoms, and more co-medications and antidepressants predicted weight gain after antipsychotic treatment
		Haloperidol = +3 kg	
		Risperidone = +4 kg	
		Olanzapine = +5−6 kg	
		**By 3 to 4 months**	
		Haloperidol = +3 to 4 kg	
		Risperidone = +6 kg	
		Olanzapine = +7−9 kg	
		**By 1 year**	After 8 weeks of treatment there was a significant increase in insulin level, insulin resistance, and glucose, cholesterol, triglyceride, and C peptide levels across clozapine, olanzapine, risperidone, and sulpiride combined but no significant difference between drugs.
		Haloperidol = +4 to 11 kg	
		Risperidone = +8 to 9 kg	
		Olanzapine = +11 to 17 kg	
		Amisulpiride = +10 kg	
		Clozapine = +10 kg	

### 7.4. Body Weight in People with SMI Who Do Not Take Antipsychotic Drugs

It has been suggested that individuals with SMI show features of metabolic syndrome before starting treatment with antipsychotic drugs [30]. However in their systematic review Foley and Morley [9] found that at baseline patients with SMI did not have a higher weight, body mass index (BMI) or waist circumference than matched controls. 

There is also some evidence that weight gain is not observed in people with SMI who do not take antipsychotic medication. Studying a cohort of 5756 French patients Limosin *et al*. (2008) [18] found higher levels of overweight and obesity in those taking antipsychotic medication compared to the general population but lower levels in those patients who were not taking medication. Padmavati *et al*. (2010) [36] also found lower BMI classifications in 51 people with enduring schizophrenia who had never taken antipsychotic medication compared to 51 healthy controls again suggesting weight gain is a feature of taking antipsychotic medication rather than SMI itself. 

### 7.5. Which Patients with SMI Gain Most Weight?

Current evidence suggests that weight gain is more rapid and severe in younger people with first episode psychosis who have had limited previous exposure to antipsychotic medication [6], those who have a lower BMI pretreatment, more negative symptoms and more co-medications and antidepressants [9]. The studies show that two of the second generation antipsychotic drugs Olanzapine and Clozapine may cause the most rapid weight gain in the early stages of treatment [9,35] and that weight gain may continue steadily for up to three years [34]. Although more research is needed to investigate further the characteristics of patients who are most vulnerable to antipsychotic related weight gain these tentative findings do nevertheless have important implications for the role of professionals who are involved in initiating and monitoring antipsychotic therapy in the early stages of treatment [37].

## 8. Why Does Antipsychotic Medication Cause Weight Gain?

There is some evidence that second generation antipsychotic medications such as Olanzapine cause people taking them to have an increased appetite and that they may promote binge eating [38]. Although the mechanisms by which this occurs are not fully understood studies have shown raised fasting serum ghrelin levels in individuals with SMI prescribed antipsychotic medication [39,40]. Ghrelin is a hormone produced in the stomach responsible for stimulating appetite and its raised levels in people with SMI may cause an increase in appetite and subsequent weight gain [38]. 

Many antipsychotic medications also have sedative properties [41] and these effects may influence the low levels of physical activity often seen in people with SMI [25,26,42]. Thus a combination of sedentary behaviour, increased appetite and poor food choices may result in an increase in weight. Furthermore loss of self confidence caused by the illness and poor self esteem resulting from weight gain [43] may maintain this vicious circle of inactivity and over eating further compounding the problem. 

### 8.1. Other Metabolic Effects of Antipsychotic Medication

In a systematic review of 77 publications Mitchell *et al*. (2013) [3] found that the prevalence of metabolic syndrome in people with SMI was 32.5% which was significantly higher than in members of the general population when matched for age. Furthermore whilst the prevalence of metabolic syndrome in the general population normally rises with age [21] Mitchell found this had only a small effect on people with SMI with the strongest influence on prevalence being the length of their illness and hence exposure to antipsychotic drugs [3]. Highest rates of metabolic syndrome were seen in those prescribed Clozapine (51.9%) and lowest in those who were not taking medication (20.2%) thus providing further evidence of the harmful metabolic side effects of second generation antipsychotic medication.

### 8.2. Metabolic Effects of Second Generation Antipsychotics on Healthy Volunteers

Sacher *et al*. (2010) [44] compared the effects of Olanzapine (10 mg/day) and Ziprasidone (80 mg/day) on whole body insulin sensitivity in 29 healthy volunteers. After 10 days exposure there was a significant decrease in whole body insulin sensitivity in the Olanzapine group but not in those taking Ziprasidone. The authors conclude that such factors should be considered when choosing a second generation antipsychotic. Albaugh *et al*. (2011) [45] conducted a double blind placebo controlled randomised crossover study to assess the metabolic effects of Olanzapine on 15 healthy volunteers. Participants were exposed to either placebo or Olanzapine (10 mg/day) for three days prior to testing. The findings showed that Olanzapine exerted some early endocrine/metabolic changes. Vidarsdottir *et al*. (2010) [46] assessed the metabolic effects of Olanzepine (10 mg/day) and Haloperidol (3 mg/day) on 14 healthy normal weight men. After 8 days the participants taking Olanzapine but not Haloperidol showed reduced fasting plasma free fatty acid concentrations and impaired glucose disposal. The finding of these studies should be interpreted with caution due to their small sample sizes and the short duration by which healthy volunteers were exposed to antipsychotic therapy. Nevertheless they do provide further evidence that some second generation antipsychotic drugs such as Olanzapine appear to have specific metabolic effects in relation to insulin production and regulation and that these effects occur rapidly after treatment is initiated. 

## 9. Interventions to Protect against Antipsychotic Induced Weight Gain

As a result of concern about weight gain and other metabolic side effects of antipsychotic medication a number of systematic reviews have recently been published which have examined the efficacy of weight management interventions for people with SMI [47,48,49]. Faulkner *et al*. (2007) [47] conducted a review of 23 studies of both pharmacological and non-pharmacological interventions to manage weight in people with schizophrenia finding that pharmacological interventions had a significant effect in the prevention of weight gain but non-pharmacological interventions such as Cognitive Behavioural Therapy had a significantly greater effect on weight reduction. Two other reviews by Alvarez-Jimenez *et al*. (2008) [48] and Caemmerer *et al*. (2013) [49] only examined the effects of non-pharmacological lifestyle interventions to promote a healthy diet and/or physical activity in people with SMI. Both reviews found that lifestyle interventions were effective in preventing anti-psychotic weight gain and promoting weight loss. Alvarez-Jimenez *et al*. [48] emphasised the importance of making these interventions a priority during the early stages of treatment to prevent weight gain.

Only two studies to date have targeted weight management interventions in younger people with first episode psychosis [50,51]. Alvarez-Jimenez *et al*. (2006) [51] randomized 61 first episode drug naïve patients to receive one of three different antipsychotics (Haloperidol, Risperidone or Olanzapine) and then randomised them again to receive either a early behavioural lifestyle intervention (EBI) focussing on diet and exercise or to a routine care control group (RCI) who were just advised about the potential side effects of antipsychotics and the need to increase exercise and limit food intake. After 13 weeks both groups had gained weight but the EBI group had gained significantly less weight than the control group (4.1 kg *versus* 6.9 kg respectively). Participants in both study groups who were prescribed Olanzapine gained significantly more weight (EBI = 6.1 kg and RCI 9.5 kg) than those on Haloperidol or Risperidone regardless of group allocation. Lovell *et al*. (2014) [51] randomized 105 first episode patients with a BMI of ≥25 (≥24 for South Asians) to either a healthy living intervention or treatment as usual. At 12 months follow up there was a small but none significant difference in BMI reduction between the groups in favor of the intervention group. 

Another systematic review by Mukundan *et al*. (2013) [52] examined the effects of switching the type of antipsychotic prescribed had on weight gain. Participants who switched from Olanzapine to Aripiprazole or Quetiapine showed weight loss, reduction in BMI as well as improvement in fasting blood sugars and lipid profiles. Although the authors caution that these findings are based on a review of only four studies.

The findings of these studies suggest that both pharmacological and non-pharmacological weight management interventions may be efficacious in helping to prevent weight gain and/or promote weight loss in people with SMI. Also for patients who gain weight rapidly in the early stages of treatment switching to an antipsychotic with a lower propensity for weight gain may be effective. Studies also suggests that non-pharmacological interventions that focus on preventing weight gain in the early stages of treatment with antipsychotic drugs [50] may be more effective than those that focus on weight loss in individuals who are already overweight or obese [51]. 

## 10. Discussion

The literature reviewed shows that individuals with a diagnosis of schizophrenia and other SMI are more likely to be overweight or obese than others in the population and that this is associated with poorer physical health [9] and reduced life expectancy [22]. A number of possible explanations for this have been explored and the evidence suggests that side effects of antipsychotic medication may be the most significant contributory factor to weight gain [6,7,8,9]. In a recent article commenting on this issue the editor of the Lancet [53] observed that in any other area of medicine an alternative treatment would be sought but for people with SMI antipsychotic medication remains the mainstay of treatment. If therefore patients with SMI must continue to receive antipsychotic medication it is imperative that professionals involved in their care do all they can to protect patients from the most serious adverse effects of the treatment. In closing we will discuss the strengths and limitations of this review and consider its implications for the future research and clinical practice. 

### 10.1. Strengths of the Review

We have conducted a broad review of the literature related to the issue of weight gain and obesity in people with SMI and the effects of this on their physical health. In order to examine the association between antipsychotic medication and obesity we explored literature about the prevalence of obesity in people taking antipsychotic medication but also compared this to studies of those with SMI who are not prescribed or have never taken antipsychotic medication. In an attempt to understand the underlying processes by which the medication causes weight gain we reviewed studies which have investigated its effects on the metabolic functioning of both people with SMI and healthy volunteers. As responsibility for the physical health care of people with SMI is often shared between a number of different professionals we have tried to review the literature in a way that is accessible and relevant to professionals both in and beyond the mental health field.

### 10.2. Limitations of the Review

As the review did not follow a systematic methodology we acknowledge that relevant literature may have been missed during our search. Also as we did not search for grey literature or papers not published in English this may have resulted in further omissions from the review. Data from studies regarding weight gain was presented descriptively and we made no attempt at meta-analysis to establish the mean weight gain across studies. Although we have commented critically on some aspects of methodological quality of research in the review we did not adopt a recognized critical appraisal tool with which to review studies.

### 10.3. Implications for Future Research

Due to methodological limitations current research is likely to have underestimated weight gain resulting from taking antipsychotic medication [6,9]. Future studies need to demonstrate more reliable findings by excluding patients who are not concordant with medication and therefore not exposed to its obesogenic effects and by not using last observation carried forward to analyze data for patients who drop out [6,9].

Studies need to attempt to predict more accurately the characteristics of patients most vulnerable to weight gain. In order to achieve this aim they should undertake thorough metabolic profiling of patients prior to commencing antipsychotic medication and then closely monitor them once treatment is initiated. This would allow current theories that younger drug naïve patients are most prone to rapid weight gain to be tested as well as further assessing the weight gain profiles of different types of medication. Studies should also continue to assess long term weight gain currently most studies only follow participants up for 12 months and no studies have followed them up beyond three years [34]. 

More research is also urgently needed to develop and refine effective pharmacological and non-pharmacological interventions to prevent weight gain and promote weight loss in people with SMI [47,48,49]. Pharmacological studies need to continue to evaluate the efficacy and safety of their products in helping people with SMI to control their weight [47]. They should also further assess the value of switching patients with rapid early weight gain to other medications with lower weight gain profiles [52]. Non-pharmacological interventions need to be further developed and evaluated. In particular there is a need for more phase specific research to investigate the efficacy of interventions to prevent weight gain in drug naïve patients who are about to commence or have only recently started treatment with second generation antipsychotic medication [6,9]. The old saying that *“prevention is better than cure”* is particularly relevant in this instance.

Research needs to investigate other possible metabolic disorders which may result from taking antipsychotic medication. The literature reviewed demonstrates a high prevalence of metabolic syndrome in people with SMI which increases their chances of developing cardiovascular disease or diabetes [20]. Yet currently no direct link between physical conditions such as diabetes and antipsychotic medication has been established. However given the abnormal metabolic responses observed in both people with SMI [3] and in healthy volunteers who have taken antipsychotic medication [44,45,46] combined with the pharmaceutical companies attempts to play down these side effects [33] clearly makes this an area worthy of further investigation. 

### 10.4. Implications for Clinical Practice

Professionals involved in the care of people with SMI who are prescribed antipsychotic medication have an important responsibility in helping them to control their weight in order to maintain good physical health [37]. Those who prescribe antipsychotic medication (particularly to drug naïve patients) should have an awareness of the characteristics of which patients are likely to gain weight most rapidly. They should use this knowledge to inform their decision about which antipsychotic medication they prescribe and avoid those with the highest weight gain profiles. Patients should be made aware of the potential side effects of different drugs and whenever possible the decision about which medication is prescribed should be a shared decision between themselves and the prescriber. This is now recommended as best practice by the UK National Institute for Health and Clinical Excellence [54]. Given its propensity to cause weight gain Clozapine should only be used for treatment resistant patients according to strict protocols when they have failed to show any clinical response to 2 or 3 other antipsychotic drugs.

Professionals who administer antipsychotic medication need to share information with patients regarding its potential to cause weight gain and the importance of healthy eating and maintaining adequate levels of physical activity to control weight gain. Weight, BMI, blood pressure and other metabolic indices should be recorded pretreatment and monitored weekly in the early stages. Any sign of rapid weight gain should trigger a review by the clinical team with consideration of whether to switch to an alternative medication with a lower weight gain profile. Behavioral weight management interventions such as the one described by Alvarez-Jimenez *et al*. (2006) [50] should be offered as standard treatment alongside antipsychotic medication [54] in order to control weight and promote good physical health. Patients should be educated about what constitutes a healthy diet and adequate levels of physical activity and they should be assisted to develop skills in budgeting and food preparation to help them maintain an appropriate calorific intake.

Patient’s prescribed antipsychotic medication over extended periods of time should continue to be monitored for any adverse effects of the treatment on their physical health. This monitoring should be a shared responsibility between the mental health and primary care team. In England and Wales all people with SMI should be offered an annual physical health check by their primary care team [55]. The aim of the health check is to screen them for emerging physical health problems including weight gain in order that appropriate management can be promptly initiated. However because of the nature of SMI and in particular the effects of negative symptoms on self-care patients will often need encouragement and support from mental health providers in order to get them to attend the physical health check. Close liaison between the primary care and mental health teams will also ensure that any issues identified in the physical health check are appropriately followed up. 

## 11. Conclusions

Despite being the cause of significant weight gain and obesity, antipsychotic medication remains the mainstay of treatment for people schizophrenia and other SMI. Its effects have been associated with poor cardio-metabolic health [9] and high levels of premature mortality [22]. Although the reasons why antipsychotic medication causes weight gain are not fully understood, the evidence we have reviewed suggests that it may interfere with the normal processes which regulate food intake and metabolism [38,39,40,44,45,46].

In this paper we have argued that research is needed to help us understand the full effects of antipsychotic medication on the physical health of those who take it but also to develop effective interventions to prevent weight gain. This is of particular importance in relation to the care of first episode drug naïve patients who are commencing treatment. We were able to identify only two studies that had evaluated such interventions with first episode patients [50,51] both gave reason for cautious optimism but more research is urgently needed. 

We have also discussed the implications of the literature for the clinical practice of professionals involved in the care of people with SMI. Good practice should include shared decision making regarding treatment options [54], careful monitoring for adverse effects of treatment and health education. Good communication between primary and secondary mental health care teams is also important to facilitate regular health checks and the early detection of signs of poor cardio-metabolic health. Routine care for patients taking antipsychotic medication should extend beyond medication and psychological support alone and should include the offer of lifestyle interventions focusing on diet and exercise [54]. 
